# Effect of feeding *Acacia nilotica* pod meal on hematobiochemical profile and fecal egg count in goats

**DOI:** 10.14202/vetworld.2016.1400-1406

**Published:** 2016-12-11

**Authors:** Jitendra Kumar Paswan, Kaushalendra Kumar, Sanjay Kumar, Chandramoni, Abhishek Kumar, Deepak Kumar, Ajit Kumar

**Affiliations:** 1 Department of Animal Nutrition, Bihar Veterinary College, Bihar Agricultural University, Patna, Bihar, India; 2 Department of Veterinary Pathology, Bihar Veterinary College, Bihar Agricultural University, Patna, Bihar, India; 3 Department of Veterinary Parasitology, Bihar Veterinary College, Bihar Agricultural University, Patna, Bihar, India

**Keywords:** *Acacia nilotica*, goats, hematology, *Haemonchus*, serum

## Abstract

**Aim:**

This study was conducted to observe the effect of feeding *Acacia nilotica* pod meal on hematobiochemical profile and gastrointestinal parasitic load in growing goats.

**Materials and Methods:**

To experiment was conducted for a period of 3-month on 24 male goats (3½ month old, average body weight [BW] 6.50±1.50 kg), distributed into four groups of six animals each. The experimental animals were fed graded level of *A. nilotica* pod meal (0%, 10%, 20% and 30%) mixed in concentrate mixture equivalent to tannin concentration of 0%, 1.91%, 3.82% and 5.73% in the total mixed ration I, II, III and IV, respectively, but *ad libitum* measured quantity of green sorghum fodder (*Sorghum bicolor*) feeding. The blood samples were collected from experimental goats during the feeding experiment for the examination of different hematological indices and serum biochemical profile to know the overall health status of animals and standard method was followed to analyze the samples. Fecal sample was collected directly from the anus of goats by inserting middle finger and kept the samples in labeled polythene bag. Further fresh sample was processed and examined by McMaster Technique for eggs per gram and oocysts per gram. It gives accurate information regarding severity of infection.

**Results:**

The feeding of babul pod meal did not address significant changes about the hematological parameters among various treatment groups. The lymphocyte count was significantly higher (p=0.07) in T_3_ group as compared to control and increase with increase in level of babul pod meal in the diet. Blood urea nitrogen (BUN) level was 4.86 and 6.59% lower in T_1_ and T_2_ group as compared to control and inversely proportional with level of supplement in ration. The decrease in BUN reflected good dietary protein metabolism happened in animals supplemented with babul pod meal. Serum creatinine level was significantly lower (p<0.01) in T_2_ group as compared to control. The creatinine level was 20.17% lower in T_2_ group as compared to control. *Haemonchus* and Coccidian egg count was significantly reduced (p<0.01) in T_2_ and T_3_ group followed with T_1_ as compared to control group.

**Conclusion:**

The metabolic status of the animal was not affected with the supplementation of babul pod meal, however, lower serum creatinine level and remarkable reduction in nematode, as well as protozoan egg count in the treatment group, showed good health impact of babul pod.

## Introduction

There is a shortage of feeds and fodders in most of the developing countries including India, which affects the productivity of the livestock. Hence, pressure on utilization of unconventional feed resources has been increasing to develop least cost rations. The majority of the livestock are fed on poor quality roughages including crop residues, tree foliage, and agro-industrial by products. The digestibility of these roughages is lower due to incriminating factors such as lignin and tannins. Livestock consuming tannin-rich diets (>5%, w/v tannin) usually develop negative nitrogen balances, lowered feed digestibility, and animal performance [1,2]. However, depending on their chemical nature and concentration in feedstuffs, tannins might be beneficial to ruminants with its positive effects such as protein sparing action, prevent bloat, and anthelmintic activity [3,4].

Babul pod are rich in protein and found abundantly in tropical countries; however, farmers are not much aware about its nutritional importance for livestock feeding specially in sheep and goat. *Acacia nilotica* pods can be used as an energy source in a concentrate mixture for ruminants and improves the efficiency of energy utilization in ruminants. They also contain all the essential amino acids in good proportions comparable to egg protein [5].

The serum biochemical and hematological features have attracted many workers to look at their indices to make clinical predictions of the health status of a particular animal [6]. However, literature available on the nutritional potential of *A. nilotica* pods and their tannin metabolites in ruminants are scanty. The nutritional status and performance of ruminants as influenced by gastrointestinal nematodes (GINs) and the grazing animals are most susceptible to GIN infection (55.48%) than stall fed animals (19.44%) due to exposure of fecal contamination of grazing land and also due to pressure on grazing land [7]. The potential of using condensed tannins (CTs) to control GINs and improve small ruminant performance in pastoral grazing systems however, many GIN species have developed resistance to anthelmintic drugs [8]. Infestation with internal parasites causes significant production losses. The use of phytochemicals is becoming preferable and may offer better control than anthelmintics to treat GINs [9].

Hence, an attempt is made to assess nutritional potential of *A. nilotica* pod meal in a total mixed ration (TMR) at different dietary level on metabolic status and gastrointestinal parasitic burden of goats under hot humid agro climatic condition of Indian state. This study will give more information about the effective utilization of unconventional feed resources, which will help in developing feeding strategies for better utilization of nutrient from tannin rich feeds by goats under tropical environment.

## Material and Methods

### Ethical approval

The study was conducted following approved guidelines of the Institutional Animal Ethics Committee with Permission No.1365/GO/R/S/L/10/CPCSEA.

### Experimental design, management, and laboratory analysis

For growth study 24 growing male goats (3½ month old, average BW 6.50±1.50 kg) for a period of 90-day were distributed into four groups of six animals each on the basis of BW in a randomized block design. All the kids were maintained in the Instruction Livestock Farm Complex, Bihar Veterinary College, Patna, India. The castrated male goats were procured from Local ICAR Institute for experimental trial. However, experimental kids were not exposed with any anthelmintic drugs and get inoculated naturally under grazing system before starting of trial. Scheduled vaccination and standard managemental practices were followed during the experiment. The experiment was conducted during monsoon season and started from July to the end of September month.

The goats were penned individually in a well-ventilated shed with cemented floor. The experimental animals were fed graded level of babul pod meal (0%, 10%, 20% and 30%) to control and three treatment groups mixed in concentrate mixture (wheat bran 32%, soya bean meal 30%, maize 35%, mineral mixture 2% and salt 1%) in the TMR I, II, III and IV, respectively, but *ad libitum* measured quantity of green sorghum fodder (*Sorghum bicolor*) of MP Chari variety developed by JNKVV, Jabalpur, India in 1985. *A. nilotica* pod was procured from locally available babul plant directly from field, dry it properly and processed as meal using grinder machine for feeding to experimental goats. Concentrate level was adjusted in such a way that proportion of concentrate intake did not exceed 50% of total dry matter intake (DMI), and thus, the concentrate to roughage ratio was maintained as 1:1. Water was provided to the animals thrice a day. The animals were fed as per the feeding standards NRC [10].

The proximate principles of the feed were analyzed by methods of AOAC [11]. The fiber fractions, acid detergent lignin, hemicellulose, and cellulose (expressed inclusive of ash) were estimated without amylase as per method of Van Soest *et al*. [12]. Phenolic constituents of babul pod meal were analyzed by method of FAO/IAEA [13] presented in Table-1.

**Table-1:** Chemical composition (%, DM) of feed stuffs used in the study.

Attributes	Concentrate mixture	*A. nilotica* pod meal	Sorghum fodder
DM	95.69	94.18	94.94
OM	90.23	94.21	86.53
CP	20.27	17.34	6.53
EE	3.12	4.15	3.63
CF	5.72	15.32	30.84
Ash	9.77	5.79	13.47
NFE	61.12	57.40	37.53
NDF	42.47	34.56	65.98
ADF	13.06	28.25	40.34
ADL	1.87	2.32	14.34
Hemi cellulose	29.41	6.31	25.64
Cellulose	11.19	25.93	26.00
Phenolic constituents			
Total phenolics	-	22.45	-
Total tannin phenolics	-	19.09	-
Non-tannin phenolics	-	3.36	-
CTs	-	1.94	-
HTs	-	17.15	-

*A. nilotica=Acacia nilotica*, DM=Dry matter, OM=Organic matter, CP=Crude protein, EE=Ether extract, CF=Crude fiber, TA=Total ash, NFE=Nitrogen free extract, NDF=Neutral detergent fiber, ADF=Acid detergent fiber, ADL=Acid detergent lignin, CT=Condensed tannin, HT=Hydrolysable tannins

### Hematobiochemical study

The blood samples were collected by puncturing the jugular vein using disposable syringes in a sterilized two set of vial, one with without anticoagulant and other with anticoagulant ethylenediaminetetraacetic acid (EDTA) from each goat at 12^th^ week of feeding experiment before the morning feeding. Blood samples with EDTA was used immediately for hematological tests such as hemoglobin (Hb) [14], packed cells volume [15], red blood corpuscles, mean corpuscular volume (MCV), mean corpuscular Hb (MCH), MCH concentration (MCHC), neutrophils, lymphocytes, basophils, monocytes, and eosinophils were performed.

Blood without anticoagulant allowed clotting and separating the serum in fresh vial and stored at −20°C for the analysis for serum glucose, total protein, albumin, globulin, urea, uric acid, blood urea nitrogen (BUN), creatinine, cholesterol, triglyceride, high-density lipoprotein, low-density lipoprotein (LDL), very LDL, serum glutamate oxaloacetate transferase (SGOT), serum glutamate pyruvate transferase (SGPT), and alkaline phosphatase. The estimation of different serum parameters were conducted using commercial kit method.

### Fecal examination

Fecal sample was collected directly through from the anus by inserting middle finger and kept the samples in labeled polythene bag. Further fresh sample was processed and examined by McMaster technique for eggs per gram (EPG) and oocysts per gram (OPG). In this method, a special counter is used for the estimation of number of EPG or larvae or OPG of feces. Counting slide having two counting chamber made up of two glass slide separated by three or four narrow transversely placed strips of glass 1.5 mm thick so that two or three space of 1.5 mm depth are obtained between the two slides. On the under surface of the upper slide an area of 1 cm^2^ is ruled over each space. The volume underneath this ruled area will therefore be 0.15 ml. Examine one chamber under microscope and multiply number of eggs or larvae under one area by 100 or two chambers and multiply by 50 to arrive at the number of EPG/OPG of feces.

### Statistical analyses

Data obtained were subjected to analysis completely randomized design with the simple analysis of variance technique [16] using Statistical Package for the Social Sciences [17]. Homogenous subsets were separated using Duncan’s multiple range test described by Duncan [18]. Differences among treatments were considered to be significant when p≤0.05.

## Results

In this study, different parameters such as hematological parameters, serum profiles, and gastrointestinal parasitic load were observed, respectively.

### Hematological parameters

The feeding of babul pod meal in total DMI affect the Hb level slightly (p=0.08) and it was fall within the normal range (Table-2). There was no significant difference (p>0.05) among various treatment group was noted, whereas numerical value of packed cell volume (PCV) in T_2_ group was higher than control. However, the MCV, MCH, and MCHC value were numerically higher in T_3_ group as compared to control. The neutrophil count was significantly higher (p<0.05) in T_2_ group as compared to control and T_3_ groups. The lymphocyte count was significantly higher (p=0.07) in T_3_ group as compared to control and T_2_ groups. The increase in lymphocyte count in T_3_ group reflect toward higher immunity of animals and it might be due to the presence of secondary metabolites in babul pod meal had synergistic effect on metabolism. However, monocyte, eosinophil and basophil count were found to be nonsignificant (p>0.05) among the various groups.

**Table-2:** Effect of feeding *A. nilotica* pod meal on whole blood parameters of goats.

Attributes	Control	T_1_	T_2_	T_3_	p value
Hb (g/dl)	10.6±0.83^ab^	10.2±0.30^a^	11.7±0.11^ab^	11.9±0.13^b^	0.088
PCV (%)	31.5±1.83	31.2±0.94	34.3±0.13	33.5±0.42	0.179
RBC (×10^6^ µL)	5.25±0.30	5.20±0.16	5.72±0.02	5.59±0.07	0.178
MCV (fL)	60.7±1.21^ab^	59.0±1.53^a^	61.3±0.80^ab^	63.7±0.66^b^	0.090
MCH (pg)	20.2±0.40^ab^	19.7±0.51^a^	20.5±0.27^ab^	21.3±0.22^b^	0.090
MCHC (g/dl)	33.7±0.67^ab^	32.8±0.85^a^	34.0±0.44^ab^	35.4±0.37^b^	0.090
Neutrophil (%)	31.0±2.08^ab^	36.7±1.20^b^	32.3±0.88^ab^	33.0±1.73^ab^	0.137
Lymphocyte (%)	54.7±2.60^ab^	51.3±1.76^a^	60.3±1.20^bc^	62.3±0.88^c^	0.007
Monocyte (%)	1.67±0.33	1.67±0.33	2.67±0.88	1.67±0.33	0.482
Eosinophil (%)	2.00±1.00	1.00±0.00	1.67±0.33	0.67±0.33	0.366
Basophil (%)	0.67±0.33	0.33±0.33	0.67±0.33	0.33±0.33	0.802

abc Values with different superscripts in a row differ significantly (p<0.05). *A. nilotica=Acacia nilotica*, Hb=Hemoglobin, PCV=Packed cells volume, RBC=Red blood corpuscles, MCV=Mean corpuscular volume, MCH=Mean corpuscular hemoglobin, MCHC=Mean corpuscular hemoglobin concentration

### Blood biochemical profile

Data on blood serum indices of goats are presented in Table-3. The glucose level numerically increasing with level of tannin increases in ration but found to be none significantly (p>0.05) variations among the treatment groups as compared to control. However, 20% babul pod meal supplemented group had 7.04% higher total protein level as compared to control group. The increase in serum total protein level indicates the more nitrogen availability at tissue level in T_2_ group as compared to control group, whereas T_2_ group had 7.97% higher globulin level as compared to control group (p=0.079). BUN level was 4.86 and 6.59% lower in T_1_ and T_2_ group as compared to control. The decrease in BUN reflected good dietary protein metabolism happened in goats supplemented with babul pod meal. Serum creatinine level was significantly lower (p<0.01) in T_2_ group as compared to control. The creatinine level was 20.17% lower in T_2_ group as compared to control. The reduction in serum creatinine level in the treatment group showed good health impact of babul pod meal. Creatinine is a waste molecule that is generated from protein metabolism and its reduction indicates retarded catabolism rate in treatment group animals. Serum calcium and phosphorus level were significantly not affected (p>0.05) with supplementation of babul pod meal as compared to control. However, babul pod meal supplementation did not affect liver metabolic function of the treatment group animals so that liver function enzymes showed unchanged pattern. There was slight reduction in total serum cholesterol level by 8.41% in the treatment group as compared to control but change was not significant (p>0.05). The slight decreased cholesterol level might be due to the presence of some bioactive molecules which may affect the lipid metabolism in animals.

**Table-3:** Effect of feeding *A. nilotica* pod meal on blood biochemical profile of goats.

Attributes	Control	T_1_	T_2_	T_3_	p value
Glucose (mg/dl)	61.0±5.4	60.8±4.4	64.1±5.9	74.5±7.4	0.339
Total protein (g/dl)	6.53±0.28^b^	6.72±0.20^b^	6.99^b^±0.28	5.32^a^±0.29	0.001
Albumin (g/dl)	3.28±0.16	3.44±0.18	3.47±0.21	2.93±0.16	0.158
Globulin (g/dl)	3.26±0.34^ab^	3.27±0.19^ab^	3.52±0.37^b^	2.39±0.30^a^	0.079
A:G ratio	1.10±0.20	1.08±0.12	1.07±0.18	1.33±0.16	0.655
Urea (mg/dl)	27.5±2.1	27.0±2.0	25.8±1.6	24.1±0.95	0.546
BUN (mg/dl)	12.3±0.94	12.0±0.92	11.5±0.74	10.8±0.42	0.547
Uric acid (mg/dl)	0.72±0.07	0.70±0.13	0.80±0.07	0.79±0.08	0.820
Creatinine (mg/dl)	1.37±0.04^b^	1.08±0.06^a^	1.14±0.02^a^	1.20±0.06^a^	0.002
Calcium (mg/dl)	10.0±0.24	11.4±0.91	11.1±0.46	11.6±0.29	0.213
Phosphorus (mg/dl)	8.09±0.60	8.25±0.29	8.02±0.13	7.94±0.16	0.933
SGOT (U/L)	179±1.5	174±1.9	181±4.2	177±3.7	0.436
SGPT (U/L)	12.0±0.94	14.7±1.5	15.2±1.0	14.3±1.0	0.258
ALP (U/L)	117±5.2	121±4.3	125±4.6	118±4.1	0.604
Cholesterol (mg/dl)	127±7.5	117±9.2	121±13.4	124±10.0	0.914
Triglyceride (mg/dl)	188±6.6	198±13.5	212±10.9	200±4.4	0.381
HDL (mg/dl)	45.9±6.2	46.7±8.1	45.5±5.2	47.0±4.6	0.998
LDL (mg/dl)	43.0±4.2	46.5±10.9	46.2±10.3	52.2±9.0	0.908
VLDL (mg/dl)	37.6±1.3	39.5±2.7	42.5±2.2	40.0±0.89	0.381

ab Values with different superscripts in a row differ significantly (p<0.05, p<0.01). A:G=Albumin:globulin, BUN=Blood urea nitrogen, SGOT=Serum glutamic oxaloacetic transaminase, SGPT=Serum glutamic pyruvic transaminase, ALP=Alkaline phosphatase, HDL=High density lipoprotein, LDL=Low density lipoprotein, VLDL=Very low density lipoprotein, T1=Treatment 1 (10% *A. nilotica* pod meal fed group), T2=Treatment 2 (20% *A. nilotica* pod meal fed group), T3=Treatment 3 (30% *A. nilotica* pod meal fed group), *A. nilotica*=*Acacia nilotica*

### Fecal examination and nutrient utilization

The result of fecal egg count during the experiment in black Bengal kids is presented in Table-4. The maximum EPG was noted in control group and minimum in 30% babul pod meal supplemented group followed with 20 and 10% supplemented group. *Haemonchus* eggs and coccidian oocysts count were significantly reduced (p<0.01) in T_2_ and T_3_ group followed with T_1_ as compared to control group. This study showed that babul pod of this region had negative effect on nematode as well as protozoan egg count and remarkable reduction was noted. This might be possible due to the presence of tannin in babul pod meal having anthelmintic properties reported by several researchers. However, the percentage efficacy for *Haemonchus* (EPG) and coccidia (OPG) was 34.17, 68.35% and 37.50, 55.43% lower in T_2_ and T_3_ group as compared to control which reflected good health impact.

**Table-4:** Effect of feeding *A. nilotica* pod meal on fecal egg count and nutrient utilization of goats.

Attributes	Control	T_1_	T_2_	T_3_	p value
Initial BW (kg)	7.62±0.74	7.55±0.83	7.85±0.81	7.63±0.69	0.993
Final BW (kg)	10.9±0.81	11.2±0.91	11.9±0.90	10.8±0.79	0.759
ADG (g/day)	36.30±2.16^a^	40.4±2.18^ab^	45.6±1.99^b^	34.6±2.23^a^	0.009
Total DMI (g/day)	357±1.84^a^	363±1.43^b^	367±1.19^b^	357±2.23^a^	0.001
Nitrogen intake (g/day)	14.7±1.01	14.6±1.72	16.0±2.29	13.5±2.76	0.860
Nitrogen outgo (g/day)	6.77±0.54	6.12±1.98	5.02±0.94	6.66±1.44	0.789
Nitrogen balance (g/day)	7.97±0.47	8.42±0.26	11.0±3.23	6.89±1.33	0.488
*Haemonchus* (EPG)	658.3±71.2^c^	433.2±21.1^b^	208.4±15.4^a^	125.0±11.2^a^	0.001
Coccidia (OPG)	1533.2±238.9^c^	958.3±49.0^b^	683.3±66.7^ab^	341.7±39.6^a^	0.001

abc Values with different superscripts in a row differ significantly (p<0.05, p<0.01). BW=Body weight, ADG=Average daily gain, DMI=Dry matter intake, EPG=Eggs per gram, OPG=Oocysts per gram

In this study, babul pod meal at moderate level (20% of total DMI) did not hamper voluntary feed intake in growing goats rather it improves the feed intake. There was no significant changes (p>0.05) found statistically in respect of nutrient digestibility; however, it was numerically higher in T_2_ group which might be due to better effect of tannin rich feed supplement on nutrient digestibility if use it in moderate level but at higher level of feeding it reduces the digestibility of nutrients. All the animals were in positive nitrogen balance. However, lower fecal N losses observed in goat fed babul pod meal may indicate that some tannin-protein or tannin-ammonia complexes are dissociated in the GI tract, resulting in increased absorption of N. The final BW after 90 days of experiment was also statistically (p>0.05) similar among the groups. However, final BW and average daily gain were 9.83% and 25.48% higher in T_2_ group as compared to control group, respectively. The increase in digestibility of nutrients in 20% babul pod meal supplemented groups might be due to positive effect of it on gastrointestinal tract health.

## Discussion

The above results indicated that goats supplemented with graded level of tannin rich feed (babul pod meal) had positive effect on hematological indices, serum profile and reduction in gastrointestinal parasitic load in growing goats. Our result agreed well with the finding of Dey *et al*. [19] reported no effect on Hb, PCV, glucose, serum proteins and serum enzymes in lambs supplemented with graded levels (0-2%) of CT from *Ficus infectoria* leaves, whereas serum urea level significantly (p<0.05) lower in CT groups (1.5-2.0%). Pathak [20] did not observed any effect on Hb, PCV, glucose, serum proteins and lactate dehydrogenase in lambs fed diets having 1-2% CT supplied through a leaf meal mixture of *F. infectoria* and *Psidium guajava*. Singh *et al*. [21] found that Hb and PCV levels were found to be highest (p<0.05) in treated group; however, the PCV values were found to be statistically non-significant in goats fed on multinutrient block supplementation with and without tanniferous leaf meal mixture.

Olafadehan *et al*. [22] reported that PCV, white blood cell (WBC), lymphocytes and glucose linearly increased (p<0.05) but MCHC linearly decreased (p<0.05), while other hematological and serum parameters were similar among diets, however, dietary tannin intake was negatively correlated to BUN. Dietary tannin intake was negatively correlated to PCV, MCHC, WBC, lymphocyte, urea, and glucose. Uguru *et al*. [23] found that there were significant (p<0.05) differences in the parameters as total protein, BUN and creatinine measured in this experiment and the finding of the present study was an agreement with the above report. Mousa and Shetaewi [24] inferred the values of total protein and their fractions, urea, cholesterol, glucose, creatinine, SGOT, and SGPT concentrations were within the normal range in ewes fed on Acacia. Our finding is an agreement with the work done [9,25,26].

Gastrointestinal parasites in grazing ruminants are one of the major problems and causing economic losses under field condition in tropical countries. In this study, the supplementation of *A. nilotica* pod meal improves the GI health by remarkable reduction of *Haemonchus* and coccidian egg counts. However, low line areas are prone to helminthes infestation, and this region had coccidiosis and *Haemonchus* problem as reported by field which causes heavy production losses in goat farming. Although, experimental goats were procured from the same location, grazing pattern and climatic conditions, susceptible to similar parasitic infestation reported in this study. Kahiya *et al*. [27] studied on the effects of *Acacia karoo* and *A. nilotica* diets on *Haemonchus contortus* infections in goats and they found a significant decrease in the fecal egg counts. Tannin could complex with nutrients and inhibits nutrient availability for larval growth or decrease GI parasites metabolism directly through inhibition of oxidative phosphorylation, causing larval death and also observed inhibition of the electron transport system. The mechanisms of tannin toxicity on nematodes are unknown but it is postulated that they may impair vital processes such as feeding and reproduction of the parasite or may bind and disrupt the integrity of the parasites cuticle [28]. In the presence of CT, dietary N is partitioned toward feces probably due to a lesser proteolysis in the rumen and consequent reduction in ammonia production. The microorganisms present in rumen might have some adaptive mechanism, which enables them to degrade hydrolysable tannins (HTs) faster or decrease activity of HTs through methylation of phenolic hydroxy groups, and these microbes might be capable of working efficiently in high concentrations of tannins [2].

The effect of foliages containing CTs on GI parasites the numbers of strongyle eggs and coccidian oocysts in the feces were reduced almost 4 times in the goats fed tree foliages as compared to grasses [29]. Max [30] revealed that dietary inclusion of quebracho extract as source of tannin appreciably reduced fecal egg counts in *H. contortus* infected sheep and goats. Supplementation of CT could be used as an alternative sustainable method to control *H. contortus* and maintained health status and performance of goats in the face of parasitic challenge [9,21]. The use of phytochemicals (CT) is becoming preferable and may offer better control than anthelmintics to treat GINs. Dietary supplementation of CT through tropical tanniferous tree leaves/leaf meal mixture at low to moderate level (1-2% of DMI) was found to be effective against different developmental stages (eggs, larvae, and adult) of GIN and decreased GI parasitic load in ruminants [8]. CT from *B. pulchella* showed anthelmintic activity, affected egg viability and reduced pasture contamination, which led to the reduced infection of the animals by nematodes [31]. Condensed tannins, inhibit the life cycle of coccidia as evidenced by decreased sporulation of the oocysts of *Eimeria tenella*, *Eimeria Maxima*, and *Eimeria acervulina* [32]. The mode of action of CTs was suspected to be penetration of the wall of the oocyst and damage to the cytoplasm since the tannins could inactivate endogenous enzymes responsible for the sporulation process. This was further supported by the appearance of abnormal sporocysts in oocysts [32,33]. This report is also an agreement with the above finding as remarkable reduction in fecal egg counts of *Haemonchus* and coccidian oocytes in growing goats fed on babul pod meal of this region particularly. The reduction of FECs was directly proportional to the concentration of tannin in the ration.

## Conclusion

The results indicated that feeding of tannin rich feed (*A. nilotica* pod meal) did not affected the metabolic status of the animal, however, higher serum total protein level in 20% fed group, indicates more nitrogen availability at tissue level and reduction in serum creatinine level in treatment group showed good health impact of babul pod meal on goats, however, remarkable reduction in fecal egg count showed better GI health with better utilization of nutrients in goats fed under tropical conditions.

## Authors’ Contributions

JKP: Performed all work as a part of his thesis dissertation programme. KK: Design of the experiment, technical help in executing the research, data analysis, writing and correction of manuscript. SK: Correction of manuscript. C: Technical help and correction of manuscript. AK: Data analysis and correction of manuscript. DK: Helps in doing hematobiochemical parameters and correction of manuscript. AK: Helps in doing fecal parameters and data analysis. All the authors read and approved the final manuscript.
